# Liver failure due to relapsed myeloma and hepatic iron overload

**DOI:** 10.3332/ecancer.2020.1028

**Published:** 2020-04-30

**Authors:** Chakra P Chaulagain, Adalberto Gonzalez, Isaac Goldszer, Luis Caraballo, James E Hoffman, Leah Elson, Maria-Julia Diacovo

**Affiliations:** 1 Department of Hematology and Oncology, Maroone Cancer Center, Myeloma and Amyloidosis Program, Cleveland Clinic Florida, Weston, Florida, USA; 2 Department of Gastroenterology and Hepatology, Cleveland Clinic Florida, Weston, Florida, USA; 3 Department of Neurology, Medical University of South Carolina, Charleston, SC, USA; 4 Department of internal medicine, Kendall Regional Medical Center, Kendall FL, USA; 5 Sylvester Comprehensive Cancer Center at University of Miami, Amyloidosis and Myeloma Program, Miami, USA; 6 Department of Pathology, Cleveland Clinic Florida, Weston, Florida, USA

**Keywords:** multiple myeloma, iron overload, liver dysfunction, liver failure

## Abstract

Multiple myeloma is a hematologic malignancy that classically manifests with hypercalcaemia, renal insufficiency, anaemia and lytic bone lesions. Liver dysfunction in multiple myeloma is a lesser known complication that occurs through biliary obstruction, liver infiltration by plasma cells, amyloid/light chain deposition or due to liver injury from medications. Although transfusion-related hepatic iron overload—leading to significant liver disease—is a recognised complication in certain hematologic malignancies, little is known about transfusional iron overload in patients with multiple myeloma. We present a case of a 49-year-old female with relapsed/refractory multiple myeloma who presented with rapid onset liver failure, due to both iron deposition and malignant plasma cell infiltration of the liver as a terminal event. A review of the literature on hepatic complications in multiple myeloma patients is presented.

## Introduction

Multiple myeloma (MM) is an incurable hematologic malignancy of plasma cells. It can present with hypercalcaemia, renal failure, anaemia and/or lytic bone lesions or fractures. In general, liver involvement in myeloma is a rare event. However, direct infiltration of the liver leading to intrahepatic cholestasis, or involvement of large bile ducts leading to obstructive jaundice, has still been described.

It is biologically probable that the most frequent mechanism of liver dysfunction in multiple myeloma is related to the proteotoxicity from light chain production of the malignant plasma cells. This light chain production, within the bone marrow, may result in AL amyloid or monoclonal light chain deposition disease of liver. In addition, various anti-plasma cell therapies employed for the treatment of myeloma can infrequently cause drug-induced liver injury. Although transfusion-related hepatic iron overload and clinically significant liver disease is a recognised complication in hematologic malignancies (particularly myelodysplastic syndrome) [1], transfusional iron overload has not been previously described in patients with multiple myeloma.

Herein, we describe a case of a 49-year-old female, with relapsed multiple myeloma, who presented with liver failure—due to both iron deposition and malignant plasma cell infiltration of liver—and subsequently died shortly after diagnosis. We also report on existing literature surrounding possible mechanisms of hepatic complications in multiple myeloma patients receiving treatments.

## Case presentation

A 49-year-old female presented to the Emergency Department with profound fatigue, progressive jaundice and diffuse pruritus. Her medical history was significant for a 4-year history of relapsed, refractory international staging system stage III, IgG kappa MM. Previous treatments for MM included: 1) cyclophosphamide, bortezomib and dexamethasone (CyBorD), followed by 2) lenalidomide, bortezomib and dexamethasone, with consolidation hematopoietic stem cell transplantation after high dose melphalan 200 mg/m^2^ (MEL 200) and finally 3) lenalidomide regimen, initiated in 2013, for maintenance.

The patient relapsed in 2016 and was initially treated with: 1) carfilzomib, pomalidomide and dexamethasone, followed by 2) daratumumab, pomalidomide and dexamethasone and finally 3) a regimen of bortezomib, dexamethasone, cisplatin, doxorubicin, cyclophosphamide and etoposide. Most recently, bendamustine and dexamethasone was used as a salvage regimen, employed as the last treatment, 4 weeks prior to the presentation.

In the several months prior to presentation, the patient had become transfusion-dependent, requiring both packed red blood cells and platelets, approximately twice per week. At the time of emergency admission, her vital signs were within normal limits. Her physical exam was remarkable for dry mucous membranes, profound jaundice with skin and scleral icterus, diffuse abdominal tenderness, and hepatosplenomegaly. Relevant laboratory findings are summarised in Table 1. She had normocytic anaemia with haemoglobin (10 g/dL), decreased platelets (40,000/mcL), elevated total bilirubin (49.5 mg/dL), elevated direct bilirubin (30 mg/dL), normal aspartate transferase (AST) (18 U/L), elevated alanine transferase (ALT) (51 U/L), normal alkaline phosphatase (80 U/L), normal albumin (3.8 g/dL), normal international normalised ratio (INR) (1.3), elevated ferritin (15,037 ng/mL) and elevated iron (206 mcg/dL)—iron saturation and TIBC were not calculable.

**Figure 1.**
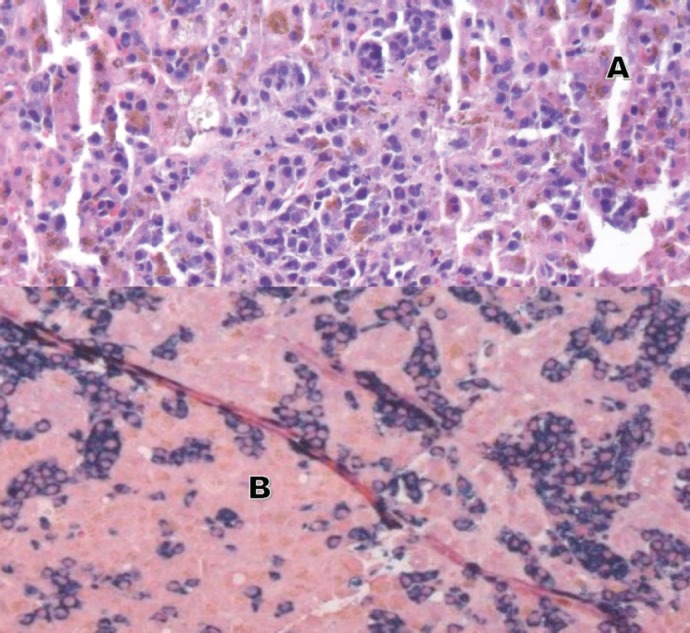
Liver biopsy (A and B). A: H&E 20×, periductular clusters of malignant plasma cells with features of plasmablasts. In addition, increased iron deposits in hepatocytes and Kupffer Cells. B: kappa and lambda CISH 10x, plasma cells with monotypic kappa staining pattern.

**Figure 2.**
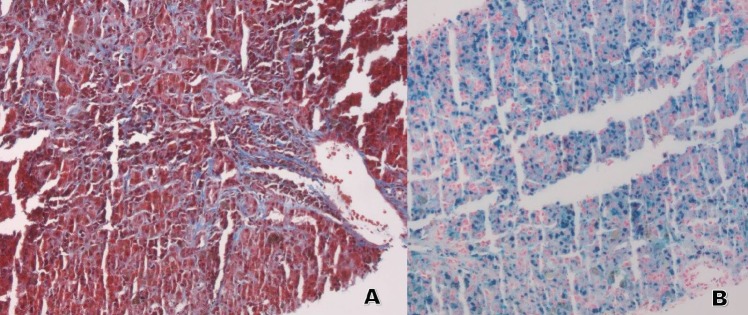
Liver biopsy. (A and B). A: Trichrome stain 10×: the stain highlights the presence of fibrosis around the malignant plasma cells. B: Iron Stain 10×: increased intracytoplasmic iron deposits in hepatocytes and Kupffer Cells.

Laboratory testing for hepatitis A, B and C, an autoimmune panel, haemolysis evaluation, and toxicology screen were unremarkable. An ultrasound of the liver demonstrated increased hepatic echotexture, but no biliary ductal dilation or hepatic nodules. A transjugular hepatic biopsy was performed to determine the etiology of liver dysfunction. The biopsy demonstrated infiltration of bile ducts and ductules by clusters of atypical plasma cells with features of plasmablasts (Figure 1A and B). A trichrome stain highlighted peri-cellular fibrosis around the infiltrate (Figure 2A). In addition, increased deposits of iron in the hepatocytes and Kupffer Cells were noted, consistent with secondary iron overload (Figures 2B).

The observed liver failure was primarily attributed to malignant infiltration by relapsed myeloma and secondarily contributed by transfusional iron overload. The patient received symptomatic and supportive therapy with ursodeoxycholic acid, diphenhydramine and transfusions, as needed. Given her poor prognosis, goals of care were discussed, and she elected for palliative care/hospice. She died a week after the discharge from the hospital.

## Discussion

Pathologic liver involvement, presenting concurrently with multiple myeloma at the time of diagnosis, is an uncommon finding. This type of presentation is often described in imaging studies as multiple nodular hepatic lesions mimicking metastatic tumours. It is often associated with poor prognosis, despite treatment efforts [2–4]. Infiltrative liver disease, due specifically to malignant infiltration by plasma cells, is also rare at the time of diagnosis, unless the presentation is due to an aggressive form of multiple myeloma manifesting as primary plasma cell leukemia [5, 6].

In contrast, diffuse hepatic infiltration, by malignant plasma cells, is much more frequent and has historically been described with an incidence rate up to 40% in an autopsy series before the discovery and use of novel agents for management of multiple myeloma [7]. Today, in the era of novel agents, the exact incidence of hepatic dysfunction, secondary to myeloma, is unknown. However, rather than *direct* hepatic plasma cell infiltration, the majority of cases reported in the literature are either secondary to hepatic amyloid deposition by systemic AL amyloidosis or, less commonly, due to monoclonal immunoglobulin light chain deposition disease of the liver [8–10].

Approximately, 10%–15% of multiple myeloma patients present with AL amyloidosis at the time of diagnosis, or will develop AL amyloidosis during the course of treatment [11, 12]. Furthermore, liver involvement in patients with systemic AL amyloidosis is seen in approximately 25% of patients [11, 12]. Amyloidosis of the liver causes infiltrative liver disease, with associated elevation of alkaline phosphatase levels and hepatomegaly. However, in these cases, severe elevation of bilirubin and jaundice is uncommon. To determine liver involvement in patients with AL amyloidosis, liver size (as determined by ultrasound) greater than15 cm in the absence of congestive heart failure, or alkaline phosphatase levels greater than 1.5 times the maximum normal values, can be used as disease indices [11, 12]. The diagnosis of amyloidosis is made by biopsy of an involved organ, or a surrogate site (e.g., abdominal fat pad, rectal mucosa or labial salivary glands)—subsequent visualisation of amyloid, with Congo red staining under polarised light, will demonstrate the characteristic apple-green birefringence. Isolated cases of jaundice, as a result of the obstruction of larger bile ducts by solid lesions from multiple myeloma, have also been described both at the time of diagnosis or at the time of relapse.

It is important to recognise that treatment-related factors may also play a role in liver dysfunction in patients with MM. For instance, anaemia is a common, concomitant finding in multiple myeloma. Additionally, anti-myeloma chemotherapy can cause severe anaemia, requiring red blood cell transfusion in some patients. This may be especially pertinent when myeloma becomes either refractory to treatment, or each remission period becomes shorter in duration as the disease becomes increasingly resistant to treatment. Transfusion-related iron overload, causing significant hepatic iron deposition, is a well-known complication in patients with myelodysplastic syndrome or haemoglobinopathies. However, red blood cell transfusion-related iron overload, causing hepatic iron deposition, is not yet well understood and has not been reported in multiple myeloma.

In this case report, it is believed that the frequent need for red blood cell transfusions, over the course of several months, contributed to the patient’s hepatic iron overload. Unfortunately, due to absence of baseline iron studies and genetic testing for hereditary hemochromatosis (HFE gene), genetic predisposition to iron overload, due to underlying HFE gene mutation, cannot be definitively ruled out. To the knowledge of the authors, there is no current literature on the treatment of multiple myeloma in patients with known history of hereditary hemochromatosis, or secondary iron overload due to red blood cell transfusion. Therefore, the authors recommend collecting both baseline iron saturation and ferritin levels. If elevated ferritin and elevated iron saturation are found in patients with multiple myeloma, suspicions should be directed to transfusional iron overload or pre-existing hereditary hemochromatosis. A restrictive transfusional threshold, and the use of iron chelating agents, should be considered to prevent deposition of iron to visceral organs in patients with documented iron overload.

Treatment outcomes in multiple myeloma patients continue to improve with the use of immunomodulatory agents (IMiD) (e.g., thalidomide, lenalidomide and pomalidomide), proteosome inhibitors [(PI) e.g., bortezomib, carfilzomib, ixazomib], and more recently the use of immunotherapy (e.g., daratumumab and elotuzumab). Although rare, all approved IMiD therapies, including thalidomide, lenalidomide and pomalidomide, have been associated with severe hepatotoxicity during the treatment of multiple myeloma [13–15]. Similarly, all proteasome inhibitors have also been reported to exhibit an association with reversible liver dysfunction. For instance, bortezomib has been described to cause reversible liver dysfunction with liver function returning to normal after discontinuation [16]. Carfilzomib has been described to cause one non-fatal, but serious, case of liver failure, despite bortezomib and lenalidomide therapy being well-tolerated, previously [17]. Approximately, 6% of patients treated with ixazomib plus lenalidomide/dexamethasone, in relapsed/refractory multiple myeloma, exhibit liver dysfunction. As such, a reduced ixazomib dosage of 3 mg (as opposed to standard 4 mg) is recommended in patients with existing moderate‐to‐severe hepatic impairment [18].

Immunotherapy represents a relatively new class of therapy available to treat multiple myeloma. Both daratumumab and elotuzumab have not yet been reported to be associated with serious hepatotoxicity or reactivation of latent hepatitis B viral infection. However, monoclonal antibody therapies, like rituximab, can cause serious hepatitis B virus reactivation, leading to fulminant hepatitis. Therefore, screening and management of latent Hepatitis B virus is recommended prior to rituximab therapy for B cell malignancies. Finally, physicians should also be aware of paraprotein interference in total bilirubin assays, which may lead to spuriously elevated total bilirubin, but normal levels of direct bilirubin in myeloma patients [19, 20]. Spurious hyperbilirubinemia from paraprotein interference may contribute to a clinical dilemma and lead to unnecessary testing. If artifactual elevation of total bilirubin is suspected, the laboratory should measure total bilirubin using alternative methods.

In conclusion, patients with myeloma and concomitant significant liver dysfunction, or liver failure, present difficulty in management. If the liver dysfunction becomes irreversible, delivering effective anti-myeloma therapy will not be possible, adversely affecting patient survival. Importantly, hepatotoxicity and rare cases of liver failure are potential consequences of chemotherapy in MM patients, even in patients who exhibit normal liver function tests upon diagnosis. Treatment options utilised to manage myeloma are generally considered hepato-safe, but in rare instances, may cause serious hepatotoxicity. Therefore, prompt recognition should be prioritised, as discontinuation of the offending agent can lead to recovery of liver function.

Plasma cell infiltration of the liver is a rare cause of death in the myeloma patient, either at the time of diagnosis or at the time of relapse. Aggressive presentation of multiple myeloma in the form of primary plasma cell leukaemia tend to cause liver involvement at the time of diagnosis; otherwise, liver involvement in myeloma is a late event in the course of the disease usually after multiple relapses or with development of secondary plasma cell leukaemia. Hepatic iron overload in this patient group can occur as a result of over transfusion. Therefore, monitoring for iron saturation and ferritin level are essential in order to better adopt restrictive transfusion practice and/or implement the use of iron chelation (in patients with documented iron overload) in order to mitigate iron deposition in the vital organs. Our patient was terminally sick at the time of presentation and was considered not a candidate for iron chelation therapy in the light of advanced relapsed myeloma with secondary liver involvement. Our patient presented with severe hyperbilirubinaemia and relatively normal liver enzymes. There was no evidence of intra-hepatic or extra-hepatic bile duct obstruction in ultrasound but microscopic bile ducts and ductules were infiltrated by malignant plasma cells causing intrahepatic microscopic cholestasis which must have played a central role in the severe hyperbilirubinaemia. The hepatic iron deposition may also have contributed to the degree of hyperbilirubinaemia. The presence of severe hyperbilirubinaemia with relatively normal liver enzymes in a myeloma patient may indicate malignant plasma cell infiltration of liver and iron deposition.

## Conclusion

Concurrent presentation of hepatic infiltration by myeloma and hepatic iron deposition is an uncommon event. This may be seen in a heavily pretreated relapsed myeloma patient with history of frequent transfusion. The presence of severe hyperbilirubinemia with relatively preserved liver enzymes levels and elevated iron saturation and ferritin may indicate malignant plasma cell infiltration of the liver and iron deposition. The diagnosis is established by biopsy. The prognosis is extremely poor as such patients are not candidates for iron chelation therapy, further antimyeloma therapy or liver transplantation.

## Conflicts of interest

The authors declare that they have no conflicts of interest.

## Funding declaration

There was no funding for this research.

## Figures and Tables

**Table 1.** Laboratory results.

Test name	Value	Normal Reference Range	Interpretation
Haemoglobin	10 g/dL	11.5-15.5 g/dL	Low
Platelet Count	41 k/uL	150-400 k/uL	Low
ALT	51 U/L	7-38 U/L	High
AST	18 U/L	13-35 U/L	Normal
ALP	80 U/L	32-117 U/L	Normal
Total Bilirubin	49.5 mg/dL	0.0-1.5 mg/dL	High
Direct Bilirubin	30 mg/dL	0.0-0.2 mg/dL	High
Albumin	3.8 g/dL	3.5-5.0 g/dL	Normal
Iron	206	41-186 ug/dL	High
Ferritin	15,037	13-150 ng/mL	High
Transferrin*	Not calculated	Not calculated	N/A
Hepatitis A IgM	Negative	Negative	Normal
Hepatitis B Surface Ag	Negative	Negative	Normal
Hepatitis C Ab	Negative	Negative	Normal
Acetaminophen	Undetectable	Undetectable	Normal
INR	1.3	0.8–1.2	Normal
Creatinine	0.4	0.58–0.96 mg/dL	Normal
Calcium	12.7	8.4–10.2 mg/dL	High
SPEP M spike	0.6 g/dL	0	Abnormal
LDH	154	135–225 U/L	Normal
Haptoglobin	109	31–238 mg/dL	Normal
Reticulocyte count	1.4%	0.4%–2.0%	Normal
DAT	Negative	Negative	Normal

ALT: alanine transferase; AST: aspartate transferase; ALP: Alkaline phosphatase; Ag: antigen; Ab: antibody, *Transferrin saturation is not reliable when ferritin levels >1,200 ug/L. LDH: lactate dehydrogenase, DAT: direct antiglobulin test, INR: International normalised ratio, SPEP: serum protein electrophoresis.
